# Supplementary material for: Modeling glioblastoma heterogeneity as a dynamic network of cell states
Source: Mol Syst Biol. 2021 Sep 16;17(9):e10105. doi: 10.15252/msb.202010105 (PMC8444284; doi:10.15252/msb.202010105)
Supplement: Supplementary file 6 — Source Data for Figure 5 [file MSB-17-e10105-s004.zip › Figure5A_sourcedata/GSEA_3071/hallmarks_stateI.GseaPreranked.1623742531165/HALLMARK_REACTIVE_OXYGEN_SPECIES_PATHWAY.html]

Details for gene set HALLMARK\_REACTIVE\_OXYGEN\_SPECIES\_PATHWAY[GSEA]

|  || Dataset | state13071 |
| Phenotype | NoPhenotypeAvailable |
| Upregulated in class | na\_pos |
| GeneSet | HALLMARK\_REACTIVE\_OXYGEN\_SPECIES\_PATHWAY |
| Enrichment Score (ES) | 0.43452382 |
| Normalized Enrichment Score (NES) | 1.7881635 |
| Nominal p-value | 0.017676767 |
| FDR q-value | 0.013634195 |
| FWER p-Value | 0.115 |
Table: GSEA Results Summary

  

Fig 1: Enrichment plot: HALLMARK\_REACTIVE\_OXYGEN\_SPECIES\_PATHWAY      
 Profile of the Running ES Score & Positions of GeneSet Members on the Rank Ordered List

  

| PROBE | GENE SYMBOL | GENE\_TITLE | RANK IN GENE LIST | RANK METRIC SCORE | RUNNING ES | CORE ENRICHMENT || 1 | NQO1 |  |  | 113 | 1.045 | 0.0461 | Yes |
| 2 | LSP1 |  |  | 143 | 0.930 | 0.1077 | Yes |
| 3 | MGST1 |  |  | 150 | 0.915 | 0.1747 | Yes |
| 4 | GCLM |  |  | 161 | 0.891 | 0.2387 | Yes |
| 5 | MBP |  |  | 423 | 0.574 | 0.2070 | Yes |
| 6 | PRDX1 |  |  | 447 | 0.549 | 0.2417 | Yes |
| 7 | JUNB |  |  | 454 | 0.546 | 0.2810 | Yes |
| 8 | GLRX |  |  | 485 | 0.524 | 0.3118 | Yes |
| 9 | PRDX4 |  |  | 494 | 0.519 | 0.3485 | Yes |
| 10 | PDLIM1 |  |  | 539 | 0.487 | 0.3724 | Yes |
| 11 | FTL |  |  | 564 | 0.473 | 0.4011 | Yes |
| 12 | TXNRD1 |  |  | 670 | 0.423 | 0.4028 | Yes |
| 13 | G6PD |  |  | 671 | 0.423 | 0.4345 | Yes |
| 14 | SRXN1 |  |  | 920 | 0.346 | 0.3894 | No |
| 15 | GCLC |  |  | 1064 | 0.314 | 0.3720 | No |
| 16 | SELENOS |  |  | 1287 | 0.270 | 0.3287 | No |
| 17 | ABCC1 |  |  | 1359 | 0.258 | 0.3277 | No |
| 18 | NDUFA6 |  |  | 1420 | -0.250 | 0.3293 | No |
| 19 | OXSR1 |  |  | 2036 | -0.323 | 0.1773 | No |
| 20 | NDUFB4 |  |  | 2164 | -0.346 | 0.1669 | No |
| 21 | CDKN2D |  |  | 2832 | -0.514 | 0.0143 | No |
| 22 | GPX4 |  |  | 3103 | -0.679 | -0.0120 | No |
| 23 | PRDX2 |  |  | 3462 | -1.713 | 0.0140 | No |
Table: GSEA details [plain text format]

  

Fig 2: HALLMARK\_REACTIVE\_OXYGEN\_SPECIES\_PATHWAY: Random ES distribution      
 Gene set null distribution of ES for **HALLMARK\_REACTIVE\_OXYGEN\_SPECIES\_PATHWAY**
